# Complete Genome Sequence of the Cluster B4 Mycobacteriophage Lolalove, Isolated in Charleston, South Carolina

**DOI:** 10.1128/MRA.00493-21

**Published:** 2021-07-01

**Authors:** Christine A. Byrum, Hannah Marie Rozier, Toni E. Allison, Emilia Ballou, Lauren Bergen, Reilley A. Chamness, Madison E. Davis, Mouna S. DiBenedetto, Nathaniel C. Elston, Lyric A. Graham, Keiana L. Haigh, Tessa M. Jansen, Gabrielle S. Kostur, Nicholas A. Larson, Fiona L. Lewis, Carlo Negroni, Isabella V. Rupert, Isabel S. Wood, Anastasia M. Zimmerman, Veronique A. Delesalle

**Affiliations:** a Department of Biology, College of Charleston, Charleston, South Carolina, USA; b Department of Biology, Gettysburg College, Gettysburg, Pennsylvania, USA; Portland State University

## Abstract

Lolalove, a B4 subcluster soil bacteriophage of Mycobacterium smegmatis, was isolated in Charleston, SC. It possesses a 71,111-bp linear double-stranded DNA (dsDNA) genome with 99 protein-coding genes and a GC content of 68.9%. genome BLASTn alignments indicate high sequence identity to the related B4 subcluster M. smegmatis phages BrownCNA, Mithril, and Hangman.

## ANNOUNCEMENT

**FIG 1:**
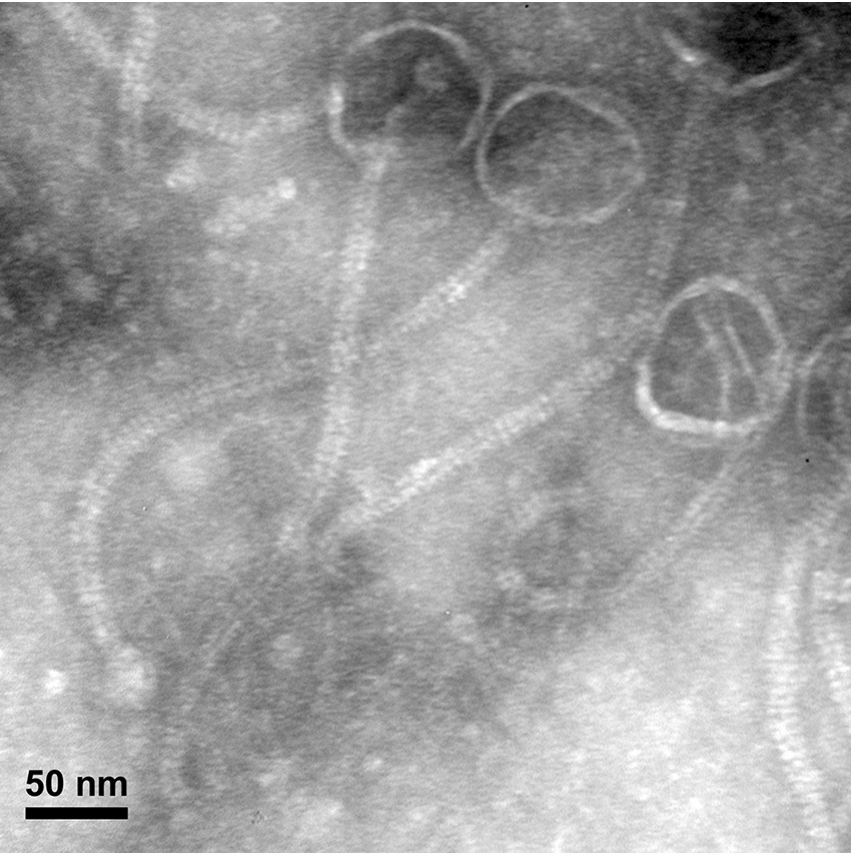
Lolalove morphology was examined using a JEOL JEM-1010 transmission electron microscope. A high-titer lysate placed on Formvar-coated copper grids was negatively stained with 1% uranyl acetate (2).

The mycobacteriophage Lolalove was discovered in damp soil at a Shell gas station in Charleston, SC (32.787021N, 79.934143W), as part of the Howard Hughes Medical Institute (HHMI) Science Education Alliance Phage Hunters Advancing Genomics and Evolutionary Science (SEA-PHAGES) (1) effort to compare genomes of novel actinobacteriophages. This virus infects Mycobacterium smegmatis mc^2^155 and was isolated using enrichment (37°C, 48 h) followed by 3 purification and amplification cycles in 7H9 top agar (see SEA-PHAGES Phage Discovery Guide for details) (2). Transmission electron microscopy revealed that this virus has *Siphoviridae* morphology with a mean capsid diameter of 76.83 nm, tail length of 329.34 nm, and tail diameter of 12.8 nm (Fig. 1).

DNA was extracted using the Promega Wizard DNA cleanup system and a sequencing library prepared with an NEBNext Ultra II library prep kit. Pittsburgh Bacteriophage Institute sequenced the genome using Illumina MiSeq technology (v3 reagents) (3) and 609,904 single-end reads (150 bp) were collected. During assembly, ∼20% of reads were from a second B4 subcluster virus. The two genomes were resolved using AceUtil (3) based on differences in contig coverage. Lolalove reads were assembled *de novo* into a single contig using Newbler v2.9 (4) and verified using Consed v29.0 (3, 5). The Lolalove genome is 71,111 bp with 1,193× coverage. The GC content is 68.9%, and the termini are circularly permuted as the contig ends overlap (3). Base 1 in the genome was selected relative to the terminase sequence (identified using DNA Master) (6) as described by Russell (3).

After sequencing, the genome was annotated using PECAAN (7), and data were subsequently transferred to DNA Master v5.22.23 (https://phagesdb.org/DNAMaster). To identify genome features and assign putative gene functions, programs used included GLIMMER v3.0 (8), GeneMark v2.5 (9), Starterator v1.1 (https://seaphages.org/media/docs/Starterator_Guide_2016.pdf), BLASTp v2.9 (10), HHpred (11), NCBI Conserved Domain Database (12), TMHMM2 (http://www.cbs.dtu.dk/services/TMHMM), TOPCONS v2 (13), Phamerator (14), ARAGORN v1.2.38 (15), and tRNAscan-SE v3.0 (16). Default settings were used in all programs.

Lolalove is a cluster B/subcluster B4 bacteriophage whose genome contains 99 putative protein-coding genes with 31 assigned functions but no tRNAs or transfer-messenger RNAs (tmRNAs) (viruses are in the same cluster if they share >50% nucleotide sequence similarity) (17). Predicted genes for structural assembly occur on the forward strand of the left arm, and as in other B4 subcluster members, no frameshift is detected in the tail chaperone protein (gp23). Putative genes on the reverse strand include gp6, gp7, gp19, gp40 to gp43, gp46 to gp60, and gp75 to gp99.

The genome similarity of Lolalove to related viruses was evaluated using tools online at https://phagesdb.org to measure whole-genome BLASTn alignment (10) and genome content similarity scores (GCSs) (18) (Table 1). Based on percent identity, Lolalove shows the highest nucleotide similarity to the B4 subcluster genomes of BrownCNA, Mithril, Hangman, Waleliano, Zemanar, and Fortunato. These mycobacteriophages were predominately isolated from sites in the southeastern United States.

**TABLE 1:** Comparison of the Lolalove genome to related B4 subcluster genomes

Phage name	GenBank accession no.	Genome size (bp)	GC content (%)	Fold coverage (×)	Location found	No. of CDSs^*a*^	% identity	% query coverage	GCS score
Lolalove	MT818419.1	71,111	68.9	1,193	Charleston, SC	99	NA^*b*^	NA	NA
BrownCNA	KT270441	71,214	68.9	1,433	Winder, GA	97	98.46	98	95.3
Mithril	MN369759	70,937	69.0	211	Louisville, KY	95	98.41	97	94.2
Hangman	MH513970	71,376	68.9	2,011	Conway, SC	97	97.87	97	97.4
Waleliano	MK524486	70,963	68.9	408	Catonsville, MD	96	97.83	97	96.9
Zemanar	JF704104	71,092	68.9	NA	Fredericksburg, VA	95	97.75	97	94.2
Fortunato	KX589269	70,679	69.0	196	Arkadelphia, AR	94	96.89	96	90.5

a CDSs, coding DNA sequences.

b NA, not applicable.

### Data availability.

The Lolalove virus is available at the Pittsburgh Bacteriophage Institute in freezer box 93/grid E3. The genome sequence and raw reads appear in DDBJ/ENA/GenBank under accession number MT818419.1 and SRA under accession number SRX9117730.
